# A case of intussusception secondary to a metastatic malignant melanoma from the nasal cavity

**DOI:** 10.1093/jscr/rjad572

**Published:** 2023-10-17

**Authors:** Yuki J Ng, Leong J Loc, Kuek S Bun, Sohail Mushtaq

**Affiliations:** General Surgery, Sarawak General Hospital, Jalan Hospital, 93586 Kuching, Sarawak, Malaysia; International Student Surgical Network Malaysia, Kuala Lumpur, Malaysia; International Association of Student Surgical Society, Cape Town, South Africa; General Surgery, Sarawak General Hospital, Jalan Hospital, 93586 Kuching, Sarawak, Malaysia; Department of Surgery, Faculty of Medicine and Health Sciences, University Malaysia Sarawak, Jalan Datuk Mohammad Musa, 94300 Kota Samarahan, Sarawak, Malaysia; General Surgery, Sarawak General Hospital, Jalan Hospital, 93586 Kuching, Sarawak, Malaysia; Department of Surgery, Faculty of Medicine and Health Sciences, University Malaysia Sarawak, Jalan Datuk Mohammad Musa, 94300 Kota Samarahan, Sarawak, Malaysia; General Surgery, Sarawak General Hospital, Jalan Hospital, 93586 Kuching, Sarawak, Malaysia; Department of Surgery, Faculty of Medicine and Health Sciences, University Malaysia Sarawak, Jalan Datuk Mohammad Musa, 94300 Kota Samarahan, Sarawak, Malaysia

**Keywords:** adult intussusception, malignant melanoma, malignancy in intussusception, epidemiology, lymphovascular invasion, small bowel resection

## Abstract

About 5% of all intussusception are found in adults, up to 90% of these have an anatomical lesion with ~50% of them are malignant. Malignant melanoma commonly metastasizes to the small bowel; however, melanoma causing intussusception is rare. We describe a 57-year-old lady with a history of surgically treated malignant melanoma in her nasal cavity who came with an ambiguous intestinal obstruction. Computed tomography reported ileal-ileal intussusception, which was surgically removed via emergency open laparotomy and bowel resection. Intraoperatively the intussusception was 110 cm from the ileo-cecal valve with multiple palpable lymph nodes. Histopathology confirmed the origin as malignant melanoma with lymphovascular invasion. Our literature review found the mean prevalence of intussusception secondary to melanoma was 6.924% (SD ± 5.155). Minimally invasive techniques are reported non-inferior to open laparotomy. We argue that the open technique can provide informed decisions for adequate resection of affected bowel and lymphatic drainage.

## Introduction

Intussusception is defined as the invagination of one segment of the bowel (the intussusceptum) into an adjacent distal segment of the bowel (the intussuscipien) [1]. Intussusception in adults is rare and is estimated at ~5% of all intussusception, and the presence of an anatomical lesion has been reported in 70–90% of cases, with 50% being malignant [3]. Malignant melanoma is one of the most common carcinomas to metastasize to the gastrointestinal tract, followed by breast and lung cancer [4]. However, malignant melanoma as a cause of small bowel intussusception is rare, reported since the 80s, but still rarely described until today [5]. We describe a case of small bowel intussusception secondary to a distant metastasis of malignant melanoma from a rare source from the nasal cavity with a literature review on the current methods of treatment. This report was written according to the CARE checklist.

## Case report

Our patient is a 57-year old lady who presented with 1 week of generalized body weakness with on and off epigastric pain associated with nausea, loss of appetite, bloatedness, not passing flatus for 3 days, and constipation for 4 days. She has a history of malignant melanoma from the left nasal cavity with metastasis to the lungs and left adrenal gland. She underwent endoscopy assisted left lateral rhinotomy and was on her third cycle of Dacarbazine before admission.

Physical examination revealed a vague, nonspecific abdominal tenderness during her initial presentation to our hospital. A nasogastric tube was inserted to decompress her stomach initially. During admission, she developed 10 episodes of vomiting clear fluids and her epigastric region started to distend. Her right lumbar and umbilical region became tender on palpation. By this time, her nasogastric tube drained ~1000 mls of greenish stomach content. Otherwise, she was able to pass motion and flatulence.

### Investigations

**Figure 1:**
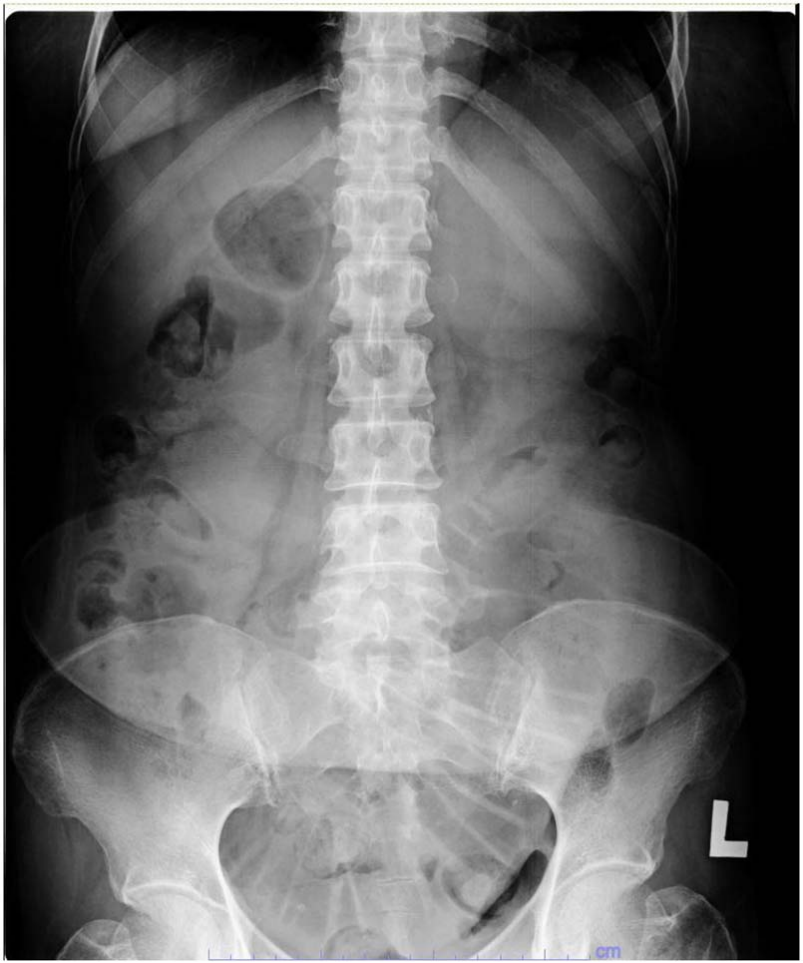
Abdominal x-ray showing a dilated small bowel with valvulae conniventes.

**Figure 2:**
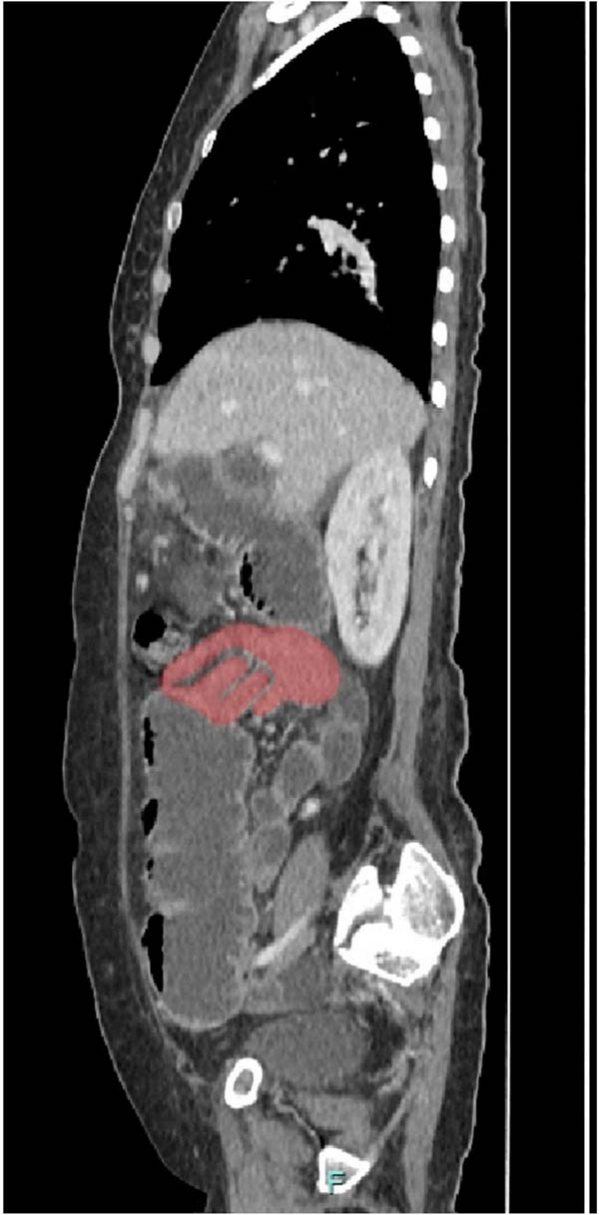
Sagittal view of CT scan. The involved bowel loops are thickened with mesenteric invagination into the intussusception. The distal small and large bowel appears collapsed.

**Figure 3:**
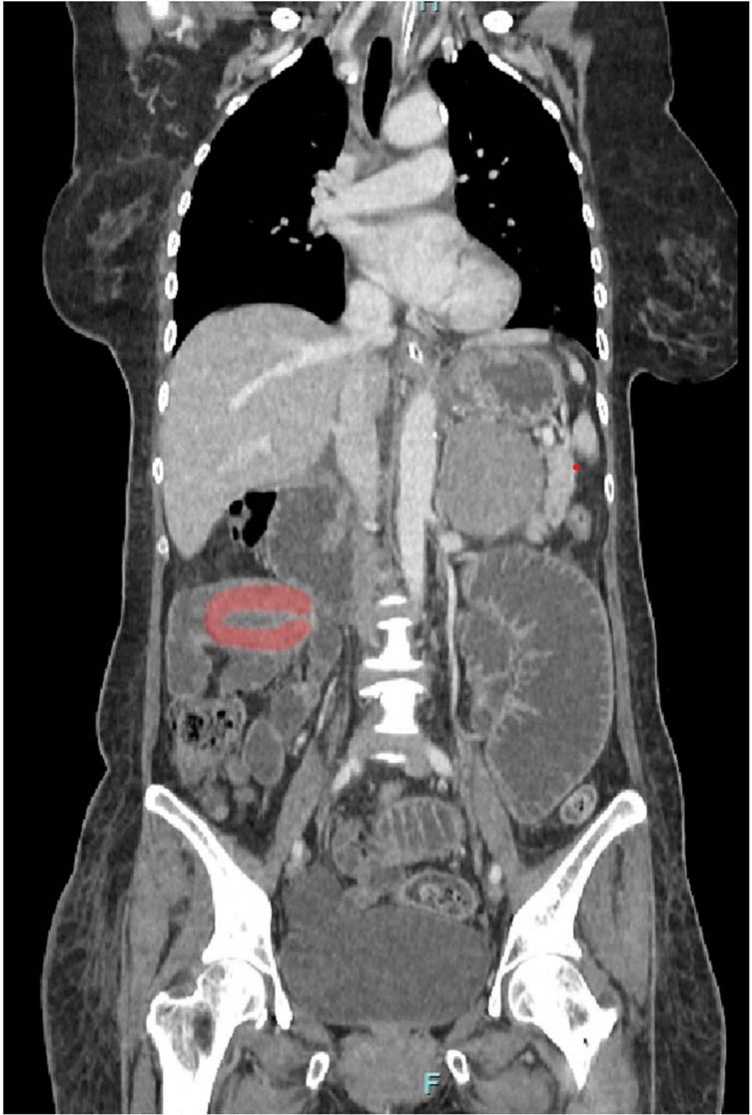
Coronal view of CT scan. Intussusception transition point is seen likely at the proximal ileum with evidence of ‘bowel in bowel’ appearance, measuring ~7.8 cm.

Her abdominal x-ray showed a dilated small bowel with a most likely transition point at the ileum and a collapsed large bowel (Fig. 1). Computed tomography (CT) of the abdomen showed an intussusception with the transition point at the proximal ileum with mesenteric invagination (Figs. 2 and 3).

### Management

**Figure 4:**
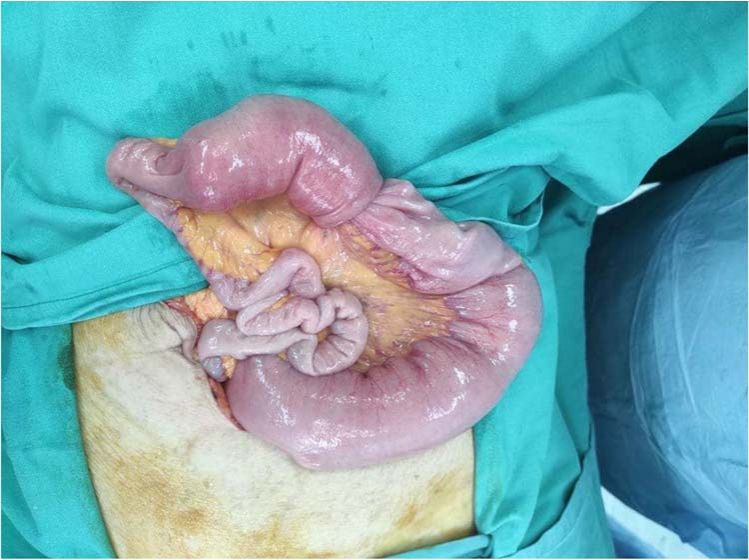
Small bowel noted to be grossly dilated proximal to the site of intussusception.

Our patient underwent an urgent midline laparotomy and small bowel resection with primary anastomosis of the small bowel. Small bowel intussusception was seen at 110 cm from the ileocecal valve. The small bowels were grossly dilated proximally from the intussusception (Fig. 4). The primary anastomosis was done with a linear stapler. Multiple lymph nodes were palpable at the surrounding mesentery of the affected small bowel at the point of intussusception. Other parts of the small bowel appeared healthy, and there were no obvious liver nodules or peritoneal nodules seen or felt.

### Histopathology

The small bowel histopathology reported an ulcerofungating malignant tumour with neutrophilic exudates and malignant cells arranged in solid sheets. The malignant cells reportedly had marked pleomorphism, vesicular nuclei, and prominent nucleoli. Malignant cells were seen to be infiltrating the mucosa, submucosa, and muscularis propria with lymphovascular invasion. The malignant cells were positive for Melan A, Sox-10, S100, and a high proliferative index of 80% from Ki67. Otherwise negative for CKAE1/AE3, LCA, chromogranin, DOG1, and desmin. Postoperatively, the patient recovered uneventfully and was discharged on postoperative Day 5.

### Follow-up

During her clinic visit, she was well with no complications, and her postoperative wound healed well. She continued with her Dacarbazine chemotherapy and oncology follow-up.

## Discussion

The clinical features in adult intussusception are often subacute or chronic, and most patients present with nonspecific symptoms that are suggestive of intestinal obstruction [6]. In addition, the classical triad of abdominal mass, tenderness, and heme-positive stools is rarely found [7]. Malignant melanoma of the nasal cavity is a rare tumour, only 1.7% of all melanomas occur in the head–neck region, usually affecting the population of 50s–70s, and almost twice more common in men [10].

### Literature search

A literature search was done in April 2023 with ‘intussusception’ and ‘adults’ for titles and abstracts on Pubmed. There were 1214 articles from the initial literature search and these articles were uploaded into RAYYAN. We included articles that showed intussusception in the adult population with cases of melanoma in the small intestines. Articles investigating the large bowel, rectum, and anal intussusception were excluded. Those articles that did not mention the pathological cause of melanoma and other causes of intussusception were also excluded. From our initial selection, 328 were identified to be retrieved for full-text screening and we further narrowed our selection to 29 articles, of which data were extracted accordingly. These articles were dated from 1953 to 2022. Extracted data are summarized in Table 1.

**Table 1:** Extracted data.

Author, year, country	Study design	Total Intussusception reported	Cases	The percentage that is melanoma	Age	Gender	Site	Imaging	Malignancy	Method of surgery
Deterling RA Jr, 1953, USA	Retrospective	40	1	2.50%	NS	NS	Small bowel	Upper GI series	Metastatic malignant melanoma	Open
Carter CR, 1989, UK	Retrospective	33	1	3.03%	NS	NS	Small bowel	NS	Metastatic malignant melanoma	Open
Goh BK, 2006, Singapore	Retrospective	60	1	1.67%	NS	NS	Small bowel	NS	Metastatic malignant melanoma	Not specified (only mentions operated)
Varban OA, 2013, USA	Retrospective	64	7	10.94%	NS	NS	Small bowel	NS	Metastatic malignant melanoma	Not specified, but mostly open in comparison to laparoscopic
Barussaud M, 2006, France	Retrospective	44	1	11.36%	70	Male	Small bowel	NS	Metastatic malignant melanoma	Open
			2		54	Male	Small bowel	CT	Metastatic malignant melanoma	Open
			3		37	Male	Small bowel	NS	Metastatic malignant melanoma	Open
			4		55	Male	Small bowel	NS	Metastatic malignant melanoma	Open
			5		69	Male	Small bowel	NS	Metastatic malignant melanoma	Open
Zubaidi A, 2006, Canada	Retrospective	22	1	9.09%	NS	NS	Small bowel	NS	Metastatic malignant melanoma	Open
			2		NS	NS	Small bowel	NS	Metastatic malignant melanoma	Open
Onkendi EO, 2011, USA	Retrospective	196	9	4.59%	NS	NS	Small bowel	CT	Metastatic malignant melanoma	Not specified (only mentions operated)
MuiÃ±os-Ruano L, 2012, Spain	Case report	1	1	100.00%	47	Female	Jejuno-jejunal	CT	Metastatic malignant melanoma	Open
Kim JW, 2018, Korea	Retrospective	77	1	1.30%	NS	NS	Small bowel	CT	Metastatic malignant melanoma	Open
El-Sergany A, 2015, USA	Case series	9	1	11.11%	65	Female	Small bowel	CT	Metastatic malignant melanoma	Laparoscopic SB resection
Kharroubi H, 2022, Lebanon	Case report	1	1	100.00%	48	Female	Ileo-ileal	CT	Metastatic malignant melanoma	Laparoscopic
Gupta V, 2011, India	Retrospective	27	1	3.70%	NS	NS	Small bowel at two sites	CT	Metastatic malignant melanoma	Open
Begos DG, 1997, USA	Retrospective	13	1	7.69%	NS	NS	Jejuno-jejunal	Upper GI series	Metastatic malignant melanoma	Open reduction and resection
Nagorney DM, 1981, USA	Retrospective	48	1	4.17%	NS	NS	Small bowel	NS	Metastatic malignant melanoma	Open
			2		NS	NS	Small bowel	NS	Metastatic malignant melanoma	Open
Azar T, 1997, USA	Retrospective	58	13	22.41%	NS	NS	Small bowel	Upper GI series/CT/USG	Metastatic malignant melanoma	Open
Kang S, 2020, Korea	Retrospective	71	1	1.41%	NS	NS	Small bowel	NS	Metastatic malignant melanoma	Not specified (only mentions operated)
Hanan B, 2010, Brazil	Retrospective	16	1	6.25%	NS	NS	Small bowel	USG/CT	Metastatic malignant melanoma	open
Butt HW, 2019, USA	Case report	1	1	100.00%	46	Male	Jejuno-jejunal	CT	Metastatic malignant melanoma	Open
Olatoke SA, 2019, Nigeria	Case report	1	1	100.00%	63	Female	Jejuno-jejunal	CT	Primary melanoma	Open
Agha FP, 1986, USA	Retrospective	25	1	8.00%	NS	NS	Ileo-ileal	NS	Metastatic malignant melanoma	Open
			2		NS	NS	Ileo-ileal	NS	Metastatic malignant melanoma	Open
Kim KH, 2014, Korea	Retrospective	33	1	3.03%	NS	NS	Small bowel	CT	Metastatic malignant melanoma	Not specified(only mentions operated)
De Monti M, 2018, Italy	Case report	1	1	100.00%	69	Male	Jejuno-jejunal	CT	Metastatic malignant melanoma	Open
De Leonardis M, 2021, Italy	Case report	1	1	100.00%	76	Female	Ileo-ileal	CT	Primary melanoma	Open
de Clerck F, 2016, Belgium	Retrospective	43	1	9.30%	55	Female	Small bowel	CT	Metastatic malignant melanoma	Open
			2		52	Female	Small bowel	CT	Metastatic malignant melanoma	Open
			3		53	Male	Small bowel	CT	Metastatic malignant melanoma	Open
			4		35	Female	Small bowel	CT/USG	Metastatic malignant melanoma	Open
Tartaglia D, 2013, Italy	Retrospective	10	1	10.00%	NS	NS	Small bowel	NS	Metastatic malignant melanoma	Not specified, but mostly open in comparison with laparoscopic
López-Tomassetti Fernández EM, 2006, Spain	Case report	1	1	100.00%	39	Female	Ileo-ileal	CT	Metastatic malignant melanoma	Open (done right hemi)
Palau T, 2005, Spain	Case report	1	1	100.00%	44	Female	Ileo-ileal	CT	Metastatic malignant melanoma	Open
Gatsoulis N, 2004, Greece	Case report	1	1	100.00%	66	Male	Ileo-ileal	CT	Metastatic malignant melanoma	Open
John F, 2005, USA	Case report	1	1	100.00%	59	Male	Jejuno-jejunal	CT	Metastatic malignant melanoma	Open

NS, not specified; CT, computed tomography; USG, ultrasonography.

Excluding case reports, throughout different cohort studies, the prevalence shows a range from 1.3 to 22.41% and a mean prevalence globally of 6.924%(SD ± 5.155). The majority of age and gender were not reported. Most articles after 1997 performed CT scans, whereas before the CT scan era, a combination of upper gastrointestinal series and ultrasound were used to diagnose intussusception with the classical ‘target and doughnut’ sign or the ‘pseudokidney’ sign [6] Out of 65 cases of intussusception caused by malignant melanoma, 1 case was reported to be a primary melanoma. Of the 29 articles, 4 articles did not specify the method of operation, 2 articles reported performing laparoscopic surgery, and 2 articles reported a mixture of both open and laparoscopic but the majority were open. The remaining 21 articles reported open surgery for all their patients.

As for imaging, a CT scan was done in most cases with an accuracy of 78–100%, and ultrasonography (USG) was the second most accurate modality thus CT scan has become the gold standard for diagnosis [11]. It may be shown on CT scan as a well-defined, intraluminal mass accompanied by a target sign, or a sausage-shaped mass, which comprises of invaginating or telescoping lead point, twisted mesentery, surrounded by thick-walled distended bowel .

From Table 1, open surgery is still the choice of management in most of the cases, but increasing case reports and studies suggest the feasibility of the laparoscopic approach [12]. Laparoscopic management of adult intussusception is safe and feasible, with a lower comprehensive complication index ie: rapid recovery with a shorter hospital stay, improved cosmetic appearance, and reduced response to surgical stress [12, 13]. Most authors agree that laparotomy is classically mandatory [14]. Open laparotomy was done in our patient, and we found tactile feedback to be a valuable tool to detect lymph node involvement.

Laparoscopy was suggested to be non-inferior as compared with open surgery. In addition, it is considered safe and feasible with adequate training and in selected cases [14]. Notably, these articles were not solely looking into intussusception secondary to malignant melanoma but purely intussusception. Reduction of intussusception has been avoided from the oncological principle of potential seeding to adjacent structures, which most surgeons practice today. En-bloc resection is still the preferred choice for surgeons globally [14, 15].

The margin of resection has been sparingly described in cases of intussusception secondary to melanoma in adults, but some authors briefly mentioned the importance of adequate proximal and distal resection from the leading point [17, 18]. We argue the potential long-term palliative benefit that a wider resection could provide in comparison with the laparoscopic method. However, studying the benefits of laparoscopy vs open method requires a randomized control trial and a very selective approach for the patient population.

## Conclusion

Intussusception secondary to malignant melanoma is rare in the adult population. It is usually caused by metastasis from a primary malignancy, in this case from an unusual occurrence of a melanoma from the nasal cavity. Although not the only method, open surgery is preferred in cases of suspected metastatic malignancies for surgical palliation. A high index of suspicion is needed for intussusception in patients who present with symptoms of subacute, ambiguous intestinal obstruction and in patients who had a history of melanoma since melanoma has a high affinity to metastasize to the small intestine.

## Acknowledgement

We are thankful to the University Sarawak Malaysia for its continuous support in research and publication.

## Conflict of interest statement

None declared.

## Funding

None declared.

## Data availability

Data are available from Y.J.N. with the permission of the patient. The data that support the findings of this study are available from the corresponding author, Y.J.N., upon reasonable request.
